# Maackiain dampens osteoclastogenesis via attenuating RANKL‐stimulated NF‐κB signalling pathway and NFATc1 activity

**DOI:** 10.1111/jcmm.15647

**Published:** 2020-09-16

**Authors:** Yuhao Liu, Weizai Zeng, Chao Ma, Ziyi Wang, Chao Wang, Shaobin Li, Wei He, Qingwen Zhang, Jiake Xu, Chi Zhou

**Affiliations:** ^1^ The First Affiliated Hospital Institute of Orthopedics Guangzhou University of Chinese Medicine Guangzhou China; ^2^ School of Biomedical Sciences University of Western Australia Perth Australia; ^3^ The First Clinical Medical College Guangzhou University of Chinese Medicine Guangzhou China; ^4^ Chinese Medicine Hospital of Zengcheng District Guangzhou China

**Keywords:** maackiain, nuclear factor of activated T cells 1, nuclear factor‐κB, osteoclast, receptor activator of nuclear factor‐κB ligand

## Abstract

Osteolytic diseases are typified by over‐enhanced formation and resorbing function of osteoclasts and have a major impact on human health. Inhibition of osteoclastic differentiation and function is a key strategy for clinical therapy of osteolytic conditions. Maackiain is a natural compound extracted from *Sophora flavescens*, which has been applied to anti‐allergic and anti‐tumour treatments. The present results showed that Maackiain could restrain receptor activator of nuclear factor‐κB ligand (RANKL)‐stimulated osteoclast formation and hydroxyapatite resorption dose‐dependently, and interrupt the structures of F‐actin belts in the mature osteoclasts. It also repressed the expressions of osteoclast‐specific genes and proteins. Furthermore, Maackiain could inhibit RANKL‐stimulated NF‐κB and calcium signalling pathways, and dampen Nuclear factor of activated T cell cytoplasmic 1 activity, protein expression and translocation into the nucleus. These results revealed that Maackiain may have a potential therapeutic effect on osteoclast‐related disorders.

## 1 INTRODUCTION

Osteolytic diseases are caused by the hyperactive differentiation and resorbing function of osteoclasts^1^ which are coupled with osteoblasts to maintain healthy bone homeostasis.^2^ Enhanced bone resorption will result in osteolytic conditions such as osteoporosis, Paget's disease and osteonecrosis.^3^, ^4^, ^5^ Especially in postmenopausal women with osteoporosis due to oestrogen deficiency, serious complications such as fragility fractures could occur under slight external force, which imposes a huge burden socio‐economically.^3^ However, the current clinical methods for preventing and treating osteoporosis are limited and often have severe side effects.^6^, ^7^ Hence, discovering novel natural compounds for osteoporosis treatments is necessary.

Bone homeostasis is maintained by coupling activities of osteoclasts and osteoblasts.^8^ As bone‐forming cells, osteoblasts are responsible for forming new mineralized bone. Osteoclasts are bone‐resorbing cells originated from hematopoietic stem cells. The differentiation and maturation of osteoclasts are inseparable from two important regulators, macrophage colony‐stimulating factor (M‐CSF) and receptor activator of nuclear factor‐κB ligand (RANKL).^9^ Via binding to RANK on the surface of osteoclast precursor cells, RANKL mediates the differentiation and resorptive function of osteoclasts.^10^ The binding of RANKL and RANK activates several signalling pathways to initiate the differentiation of osteoclast precursor cells into mature osteoclasts, importantly, NF‐κB signalling pathway and MAPK signalling pathway.^11^, ^12^ Nuclear factor of activated T cell cytoplasmic 1 (NFATc1) acts as a master transcriptional factor in the process of osteoclast differentiation,^13^ which controls the expression of downstream osteoclast‐related genes, including *Ctsk* (encoding cathepsin K), *Ctr* (encoding calcitonin receptor), *Acp5* (encoding TRAcP), and *c‐fos* (encoding c‐Fos), ultimately leading to the formation of mature multinucleated osteoclasts. Moreover, the initiation of the calcium signalling pathway can facilitate the self‐expansion and translocation of NFATc1 into the nucleus.^14^, ^15^

Maackiain is a compound extracted from the roots of *Sophora flavescens*, a traditional Chinese herb. Recent studies revealed that alkaloids from *S. flavescens* can suppress osteoclast activity and tartrate‐resistant acid phosphatase (TRAcP) activity through RANKL‐induced NF‐κB signalling pathway.^16^ Another *S. flavescens* extract named Sophocarpine has also shown its inhibitory effect on osteoclast formation and bone‐resorbing function by inhibiting the NF‐κB signalling pathway.^17^ It seems that *S. flavescens* extracts are promising agents for treating osteoclastic diseases. As reported, Maackiain can dampen human monoamine oxidase B (MAO‐B) enzyme for the treatment of disorders like Alzheimer disease, Parkinson's disease and depression.^18^ Maackiain has also been found to have functions of anti‐allergy,^19^ anti‐mosquito,^20^ anti‐tumour^21^ and pro‐apoptosis.^22^ However, it has not been reported whether Maackiain can affect RANKL‐stimulated osteoclastogenesis.

The present study investigated the role of Maackiain in osteoclast formation and function using in vitro experiments. It was found that Maackiain can restrain the differentiation and bone‐resorbing function of osteoclasts. This inhibitory effect was attributed to that Maackiain blocked RANKL‐stimulated NF‐κB and calcium signalling pathways, and reduced NFATc1 activity, protein expression and translocation into the nucleus.

## 2 MATERIALS AND METHODS

### 2.1 Materials and regents

Maackiain with a purity of ≥98% (Cat#CFN99746) was commercially obtained from Wuhan ChemFaces Technology Co., Ltd and prepared at a storage concentration of 100 mmol/L in DMSO and further diluted to working concentration in sterile PBS when used. 3‐(4, 5‐dimethylthiazol‐2‐yl)‐5‐(3‐carboxymethoxyphenyl)‐2‐(4‐sulfophenyl)‐2H‐tetrazolium) (MTS) solution was purchased from Promega. Foetal bovine serum (FBS), alpha‐modified minimum essential medium (α‐MEM), rhodamine phalloidin and Hoechst 33258 were purchased from Thermo Fisher Scientific. Primary antibodies, such as anti‐c‐Fos (Cat#2250S), anti‐integrin β3 (Cat#sc‐6617‐R), anti‐MMP9 (Cat#sc‐21733), anti‐CTSK (Cat#sc‐48353), anti‐β‐actin (Cat#sc‐47778), anti‐IκBα (Cat#sc‐371), anti‐NFATc1 (Cat#sc‐7294) and anti‐p65 (Cat#sc‐8008) antibodies, were obtained from Santa Cruz Biotechnology. Primary antibody against p‐p65 (Cat#3031S) was obtained from Cell Signalling Technology. Anti‐vinculin antibody (Cat#V9264) was purchased from Sigma‐Aldrich. Recombinant GST‐rRANKL was produced and purified as described previously.^23^ Recombinant M‐CSF (Cat#M6518) was obtained from R&D systems and diluted in PBS to a storage concentration of 1 μg/mL.

### 2.2 Isolation and purification of osteoclast precursors

Bone marrow macrophages (BMMs), the osteoclast precursor cells, were isolated from long bones of C57BL/6J female mice and then purified as described.^24^ The method was approved by University of Western Australia Animal Ethics Committee (No. RA/3/100/1601). Briefly, bone marrow was flushed out of the femur and tibia using α‐MEM. The cells were resuspended and cultured in complete α‐MEM containing 1% PenStrep (Sigma‐Aldrich), 10% FBS and 50 ng/mL M‐CSF until becoming confluent. The cells were then trypsinized for further use or cryopreservation.

### 2.3 Osteoclast differentiation and TRAcP staining

Osteoclastogenesis assay was used routinely as reported.^25^ In brief, BMMs were seeded into 96‐well plates at a density of 6 × 10^3^ cells per well. The next day, the adherent BMMs were stimulated with RANKL at a concentration of 50 ng/mL to induce osteoclastogenesis. Subsequently, various concentrations of Maackiain (0, 5, 10, 20, 30, 40 μmol/L) were added into each group of culture. Media was changed every 2 days until mature osteoclasts were formed. Then, cells were fixed with 2.5% glutaraldehyde, gently washed with PBS for twice and stained with TRAcP staining solution at 37°C for about 40 minutes. The cells of each group were photographed using an inverted light microscope, and osteoclast‐like cells with multi‐nuclei (with more than three nuclei) were scored.

### 2.4 Cell viability assay

BMMs were seeded into a 96‐well plate at a density of 6 × 10^3^ per well. The next day, different concentrations of Maackiain (0, 5, 10, 20, 30, 40 μmol/L) were added and incubated for 2 days, then 20 μL of MTS reagent was added and incubated for 2 hours in dark. MTS absorbance at 490 nm was measured using a microplate reader.

### 2.5 Hydroxyapatite resorption assay

In order to detect the bone‐resorbing function of osteoclasts, the resorption pits were observed on hydroxyapatite plates. BMMs were seeded into 6‐well plates at a density of 8 × 10^4^ per well and induced with 50 ng/mL RANKL. After mature osteoclasts were observed under a microscope, cells were detached using cell dissociation solution (Sigma‐Aldrich) and then seeded into a 96‐well plate covered with hydroxyapatite at the bottom (Corning). Cells were continued to be stimulated with 50 ng/mL RANKL in the presence or absence of 40 μmol/L of Maackiain. After 48‐hour incubation, cells were gently washed with PBS, half of the wells were stained with TRAcP solution, and the other half were washed with 10% bleach to expose resorption areas. An inverted light microscope and ImageJ software were applied to quantify the resorbing pits of hydroxyapatite as previously reported.^26^

### 2.6 Immunofluorescent staining

BMMs were seeded onto coverslips of 24‐well, and RANKL was added as described above until mature osteoclasts were formed, with the presence or absence of Maackiain (40 μmol/L). The cells were fixed with 4% paraformaldehyde and permeabilized with 0.1% (v/v) Triton X‐100 for 5 minutes, followed by being blocked with 3% BSA at room temperature for 10 minutes. The cells were incubated with anti‐vinculin or anti‐NFATc1 antibody at 4°C, followed by the incubation with a secondary anti‐mouse IgG conjugated with FITC (Thermo Fisher). Then, the cells were co‐stained with rhodamine phalloidin and Hoechst 33258 for 20 minutes. After the staining, the coverslips were mounted with the ProLong Gold Antifade Mountant and visualized using a confocal microscope (Nikon Corporation) as previously described.^27^

### 2.7 mRNA isolation and Real‐time PCR analysis

BMMs were seeded into 6‐well plates, and RANKL at a concentration of 50 ng/mL was added after the adherence, with or without different concentrations of Maackiain for 5 days. After osteoclasts were formed, TRIzol™ reagent and PureLink™ RNA Mini Kit (Thermo Fisher) were used to isolate total RNA, followed by reverse transcription of RNA into single stranded cDNA using M‐MLV reverse transcriptase with oligo‐dT primer. Real‐time PCR process was performed using the resulting cDNA, specific primers listed in Table 1 and PowerUP^TM^ SYBR Green Master Mix (Thermo Fisher). The conditions for PCR were as below: Holding stage of 95°C for 5 minutes, PCR stage (40 cycles) of 95°C for 15 seconds, and annealing at 60°C for 60 seconds, Melt curve stage of 95°C for 15 seconds, 60°C for 60 seconds and 95°C for 15 seconds. The obtained data were analysed by ΔΔC*_t_* method.

**TABLE 1:** Gene primer sequences

Genes	Forward	Reverse
*Acp5*	TGTGGCCATCTTTATGCT	GTCATTTCTTTGGGGCTT
*c‐fos*	GCGAGCAACTGAGAAGAC	TTGAAACCCGAGAACATC
*Ctsk*	GGGAGAAAAACCTGAAGC	ATTCTGGGGACTCAGAGC
*Mmp9*	CGTGTCTGGAGATTCGACTTGA	TTGGAAACTCACACGCCAGA
*Ctr*	TGGTTGAGGTTGTGCCCA	CTCGTGGGTTTGCCTCATC
*Nfatc1*	CAACGCCCTGACCACCGATAG	GGCTGCCTTCCGTCTCATAGT

### 2.8 Western blot assay

BMMs were seeded into 6‐well plates, followed by the stimulation with RANKL (50 ng/mL) for 0, 1, 3 and 5 days, in the presence or absence of Maackiain (40 μmol/L). Cells were collected and lysed with 120 μL of radioimmunoprecipitation assay buffer to extract total proteins. The proteins were loaded and separated using SDS‐PAGE, transferred onto a nitrocellulose membrane and blocked with 5% skim milk for non‐specific binding. Specific primary antibodies and horseradish peroxidase‐conjugated secondary antibodies were used for binding to proteins which were detected using enhanced chemiluminescence reagents (PerkinElmer) and visualized on an Image‐quant LAS 4000 (GE Healthcare). The band intensities were quantified with ImageJ software.

As to the protein expressions of osteoclast‐related signalling pathways, BMMs were seeded at a density of 2.5 × 10^5^ cells/well and cultured overnight. Cells were starved in serum‐free medium for 2 hours, followed by being pre‐treated with or without Maackiain (40 μmol/L) for 1 hour, and stimulated with RANKL (50 ng/mL) for various durations. Western blots were performed as above.

### 2.9 Measurement of intracellular Ca^2+^ oscillation

BMMs were seed into 48‐well plates and cultured with complete α‐MEM overnight. Cells were starved in serum‐free medium for 1 hour, followed by being pre‐treated with or without Maackiain (40 μmol/L) for 1 hour, and stimulated with RANKL (50 ng/mL). Cells were washed with HANKS balanced salt solution supplemented with 1 mmol/L probenecid and 2% FBS twice, incubated with 4 μg/mL Fluo4 solution (Thermo Fisher) for 45 minutes in the dark. Following the incubation, cells were washed again, and then left on the bench for 20 minutes, protecting from light. When the above staining steps were completed, a fluorescent inverted microscope was applied to capture the signals of calcium of individual cell within 3 minutes, and NIS‐Elements Viewer software was used to quantitatively analyse the fluorescence intensity of each cell as previously described.^28^

### 2.10 Statistic analysis

All data in present study were presented as mean ± standard deviation and statistically analysed with SPSS 17.0 software. Student's *t* tests were used for the comparison between two sets and one‐way ANOVA tests for the comparison among three or more groups. A *P*‐value ≤.05 indicates a statistical significance.

## 3 RESULTS

### 3.1 Maackiain attenuated RANKL‐stimulated osteoclastogenesis

**FIGURE 1:**
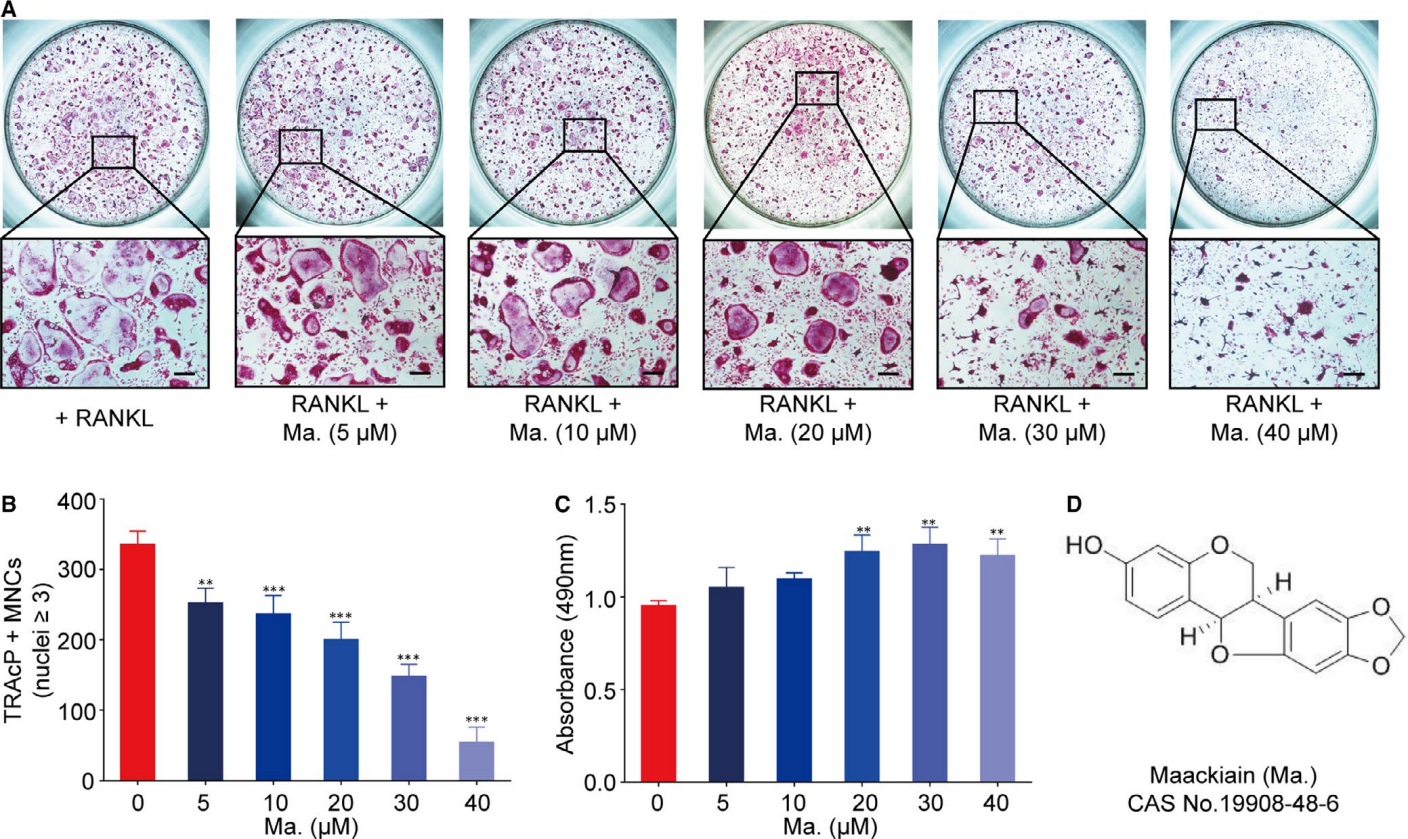
Maackiain dampened RANKL‐induced osteoclast formation. A, BMMs were seeded into a 96‐well plate at a density of 6 × 10^3^ cells/well and stimulated with RANKL in the presence or absence of Maackiain for 5 d, respectively. Representative images of 96‐well plate showed the effects of different concentrations (0, 5, 10, 20, 30, 40 μmol/L) of Maackiain. (Scale bar = 200 μm) (B) Quantification of the effect of Maackiain treatment on the number of TRAcP‐positive multinucleated cells (MNCs) (nuclei > 3) (n = 3). C, Survival of BMMs in the presence of Maackiain as assessed by MTS cell viability assay (n = 3). D, The chemical structure of Maackiain. **P < .01, ***P < .001

In order to clarify the inhibitory effect of Maackiain on osteoclast formation during RANKL induction process, gradient concentrations of Maackiain were applied to cell culture. The results in Figure 1A,B showed that Maackiain exhibited a suppressive effect on osteoclast differentiation from the concentration of 5 μmol/L. Additionally, cytotoxicity test indicated that it had no cell toxicity on BMM proliferation, as shown in Figure 1C. The chemical structure of Maackiain is shown in Figure 1D.

### 3.2 Maackiain suppressed RANKL‐induced osteoclast function

**FIGURE 2:**
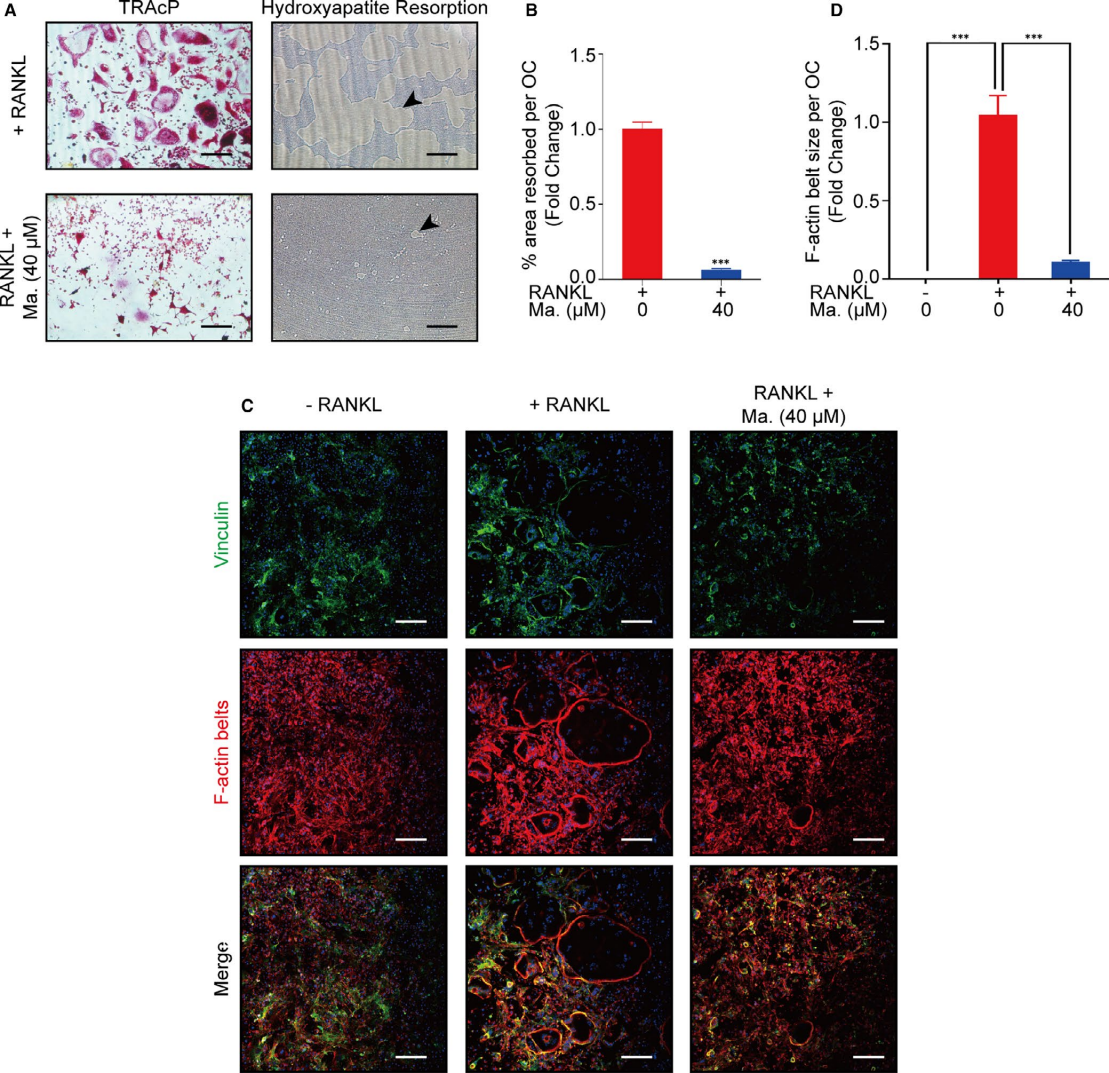
Maackiain inhibited the hydroxyapatite resorption function of osteoclasts. A‐B, 40 μmol/L of Maackiain inhibited the bone resorption function of osteoclasts, and the arrow represented the resorbed pits. C‐D, Maackiain disrupted the formation of actin belts that were co‐stained with rhodamine phalloidin (red) and vinculin (green). Scale bar = 200 μm; n = 3; ***P < .001

The resorbing pits of hydroxyapatite plate were analysed to evaluate the osteoclast function. As shown in Figure 2A,B, Maackiain of 40 μmol/L blocked osteoclastic resorption. Moreover, the rhodamine phalloidin, primary antibody of vinculin and Hoechst 33258 probe were used to co‐stain the podosomes of osteoclasts and DNA in nuclei, and F‐actin belt was presented as red, vinculin protein as green and DNA as blue. The Figure 2C,D indicated that the same concentration of Maackiain could also rupture the intact vinculin‐rich F‐actin belts when compared with the RANKL group.

### 3.3 Maackiain suppressed RANKL‐induced osteoclastic protein and gene expressions

**FIGURE 3:**
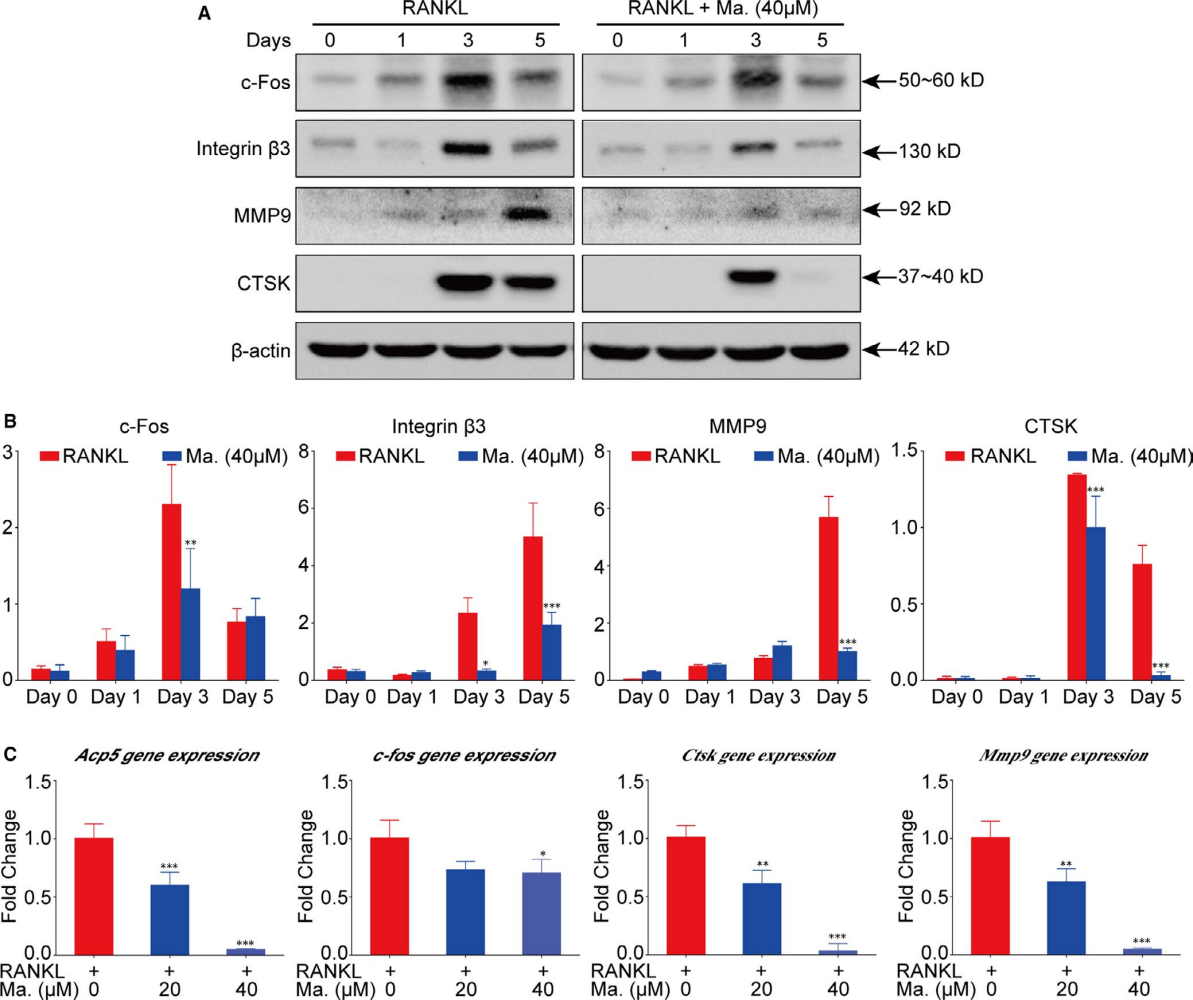
Maackiain suppressed osteoclast‐associated protein and gene expressions. A, Maackiain of 40 μmol/L inhibited c‐Fos, Integrin β3, MMP9 and CTSK protein expressions. B, Quantitative analysis of band intensities relative to the intensity of β‐actin. C, RT‐PCR results showed that Maackiain inhibited osteoclast differentiation and bone resorption‐related genes including *Acp5*, *c‐fos*, *Ctsk* and *Mmp9*. n = 3; *P < .05, **P < .01, ***P < .001

Following RANKL stimulation and Maackiain intervention for different time‐points, Western blot was utilized to detect the downstream protein expressions in the osteoclastic signalling pathways. As shown in Figure 3A,B, Maackiain decreased the expression level of c‐Fos protein at Day 3 which is associated with mature osteoclast formation, and down‐regulated bone resorption‐related proteins such as Integrin β3, MMP9 and CTSK at Day 5. Moreover, in line with the protein expressions, the results of RT‐PCR at Day 5 revealed that Maackiain also down‐regulated the expressions of osteoclastic genes such as *Acp5*, *c‐fos*, *Ctsk* and *Mmp9* (Figure 3C). These results further supported that Maackiain had a suppressive effect on osteoclast formation and function.

### 3.4 Maackiain restrained RANKL‐induced NF‐κB signalling pathway

**FIGURE 4:**
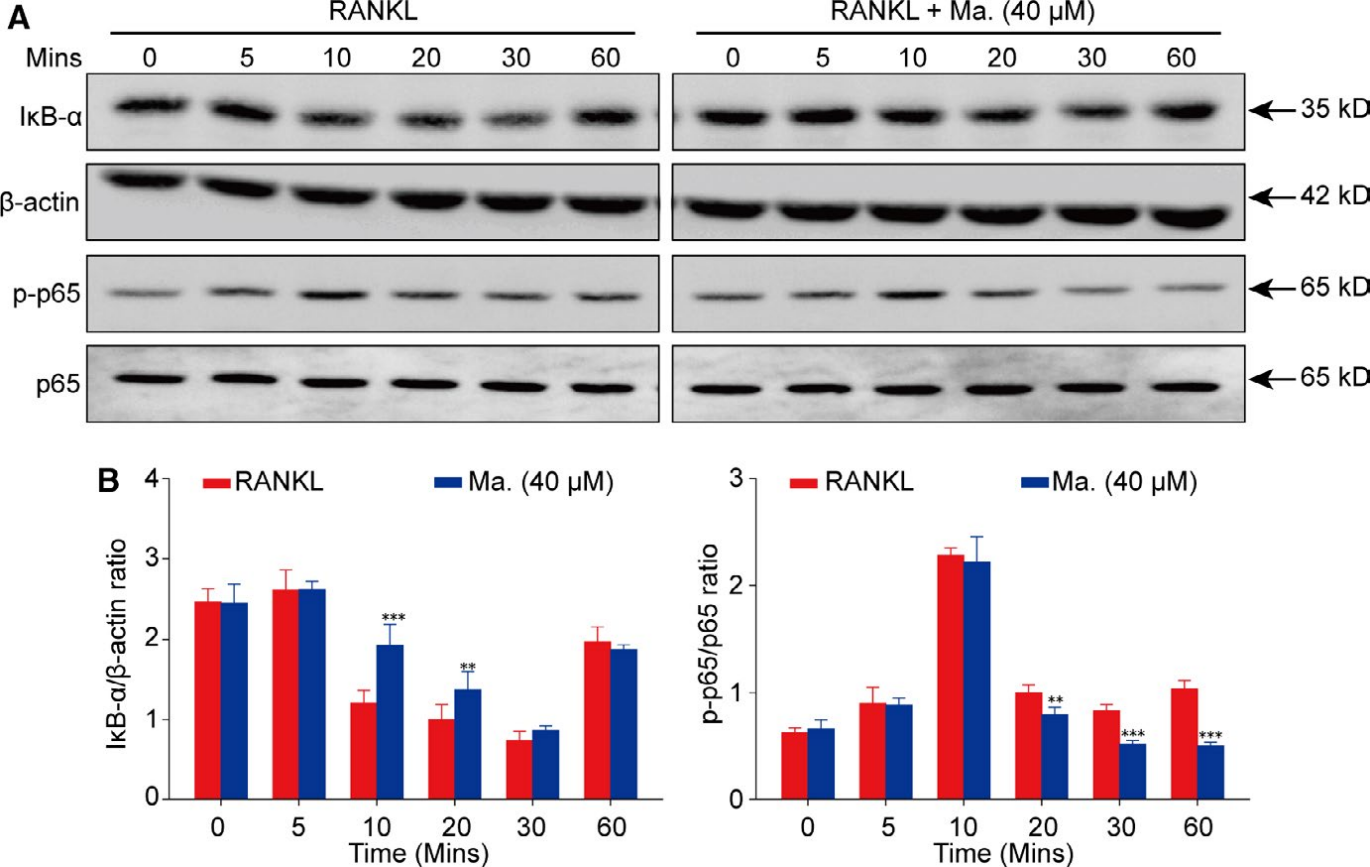
Maackiain suppressed the activation of NF‐κB signalling pathway. BMMs were stimulated with RANKL for various periods with or without 40 μmol/L Maackiain. The total protein was examined by Western blot. It indicated that Maackiain could inhibit the degradation of IκB‐α on the time‐points of 10 and 20 min, and dampened the phosphorylation of p65 since the time‐point of 20 min. n = 3; **P < .01, ***P < .001

To elucidate the molecular mechanism of Maackiain's suppressive effect, a detailed time course of the RANKL stimulation was conducted, and Western blot analysis showed that RANKL stimulation increased the degradation of IκB‐α and the phosphorylation of p65 as early as less than 30 minutes, while Maackiain had an inhibitory effect on the activation of NF‐κB signalling pathway (Figure 4).

### 3.5 Maackiain attenuated the NFATc1 activity

**FIGURE 5:**
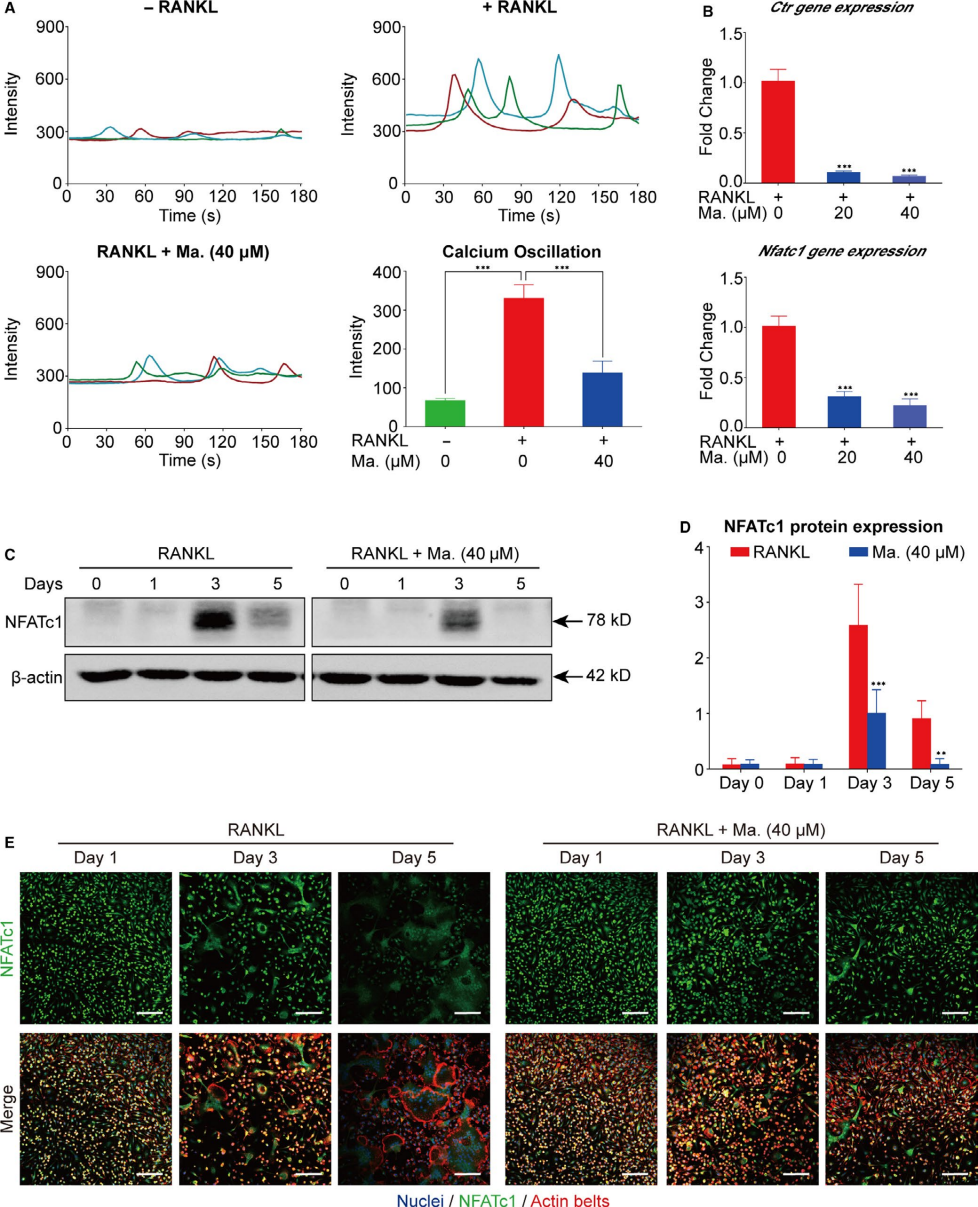
Maackiain inhibited RANKL‐induced NFATc1 activity. A, Quantitative analysis of the calcium oscillation intensity. n = 3; ***P < .001. B, Genetic expression levels of *Ctr* and *Nfatc1* were determined using RT‐PCR. n = 3; ***P < .001. C, Representative result of the Western blot to determine the NFATc1 protein level after stimulation of RANKL for 5 d with or without Maackiain. β‐actin was used as loading control. D, Quantitative analysis of the NFATc1 protein expression compared to the RANKL control group. n = 3; **P < .01, ***P < .001. E, Representative images of the immunofluorescent microscopy co‐stained for NFATc1 (green), actin belts (red) and nuclei (blue). (Scale bar = 100 μm)

As the master transcription factor during RANKL‐stimulated osteoclastogenesis, NFATc1 could be activated by Ca^2+^ signalling and then translocated into nucleus. Calcium oscillation assay indicated that Maackiain restrained the intensity of Ca^2+^ flux (Figure 5A). And the gene and protein expressions of NFATc1 had been also down‐regulated from Day 3 (Figure 5B‐D). Moreover, the co‐staining of NFATc1, nucleus and actin belts was applied to further gain insight into the nuclear translocation of NFATc1, and the observed reduction of NFATc1 both in cytoplasm and nucleus was shown in Figure 5E.

## 4 DISCUSSION

Over‐activation of osteoclasts can lead to osteolytic conditions such as Paget's disease, osteoporosis and osteonecrosis.^3^, ^4^, ^5^ The present methods of treating osteolytic diseases are limited with potential adverse effects.^6^, ^29^ A number of natural compounds extracted from Chinese medicines have shown suppression function on the maturation and bone resorption of osteoclasts.^27^, ^30^, ^31^ This study has demonstrated that Maackiain derived from *S. flavescens* has the capability of abrogating osteoclast differentiation and resorbing function, and reducing osteoclastic gene and protein expressions through attenuating RANKL‐stimulated NF‐κB signalling pathway and NFATc1 activity. These results indicated that Maackiain could be utilized as a potential therapy to prevent osteolysis in bone disorders.

The differentiation and maturation process of osteoclasts is regulated by RANKL which binds to RANK and activates downstream signalling,^10^ and the resorbing function is determined by the integrated actin cytoskeleton which has two different structures known as podosomes and sealing zone.^32^, ^33^ Our results showed that Maackiain could inhibit RANKL‐stimulated osteoclastogenesis dose‐dependently without cell toxicity. It also impaired the structures of actin podosomes in mature osteoclasts observed by immunofluorescent microscopy at Day 5, which is consistent with the reduced resorbing function of mature osteoclasts affected by Maackiain.

The NF‐κB signalling pathway activated by RANKL is one of the main signalling pathways during osteoclastogenesis. The binding of RANKL and RANK could further induce the degradation of IκB‐α and then the phosphorylation of p‐65 which is translocated into the nucleus and initiates the expressions of osteoclast‐related genes and proteins.^34^ Western blot results of a detailed time course revealed that Maackiain could attenuate NF‐κB signalling pathway through preventing IκB‐α degradation and p65 phosphorylation.

**FIGURE 6:**
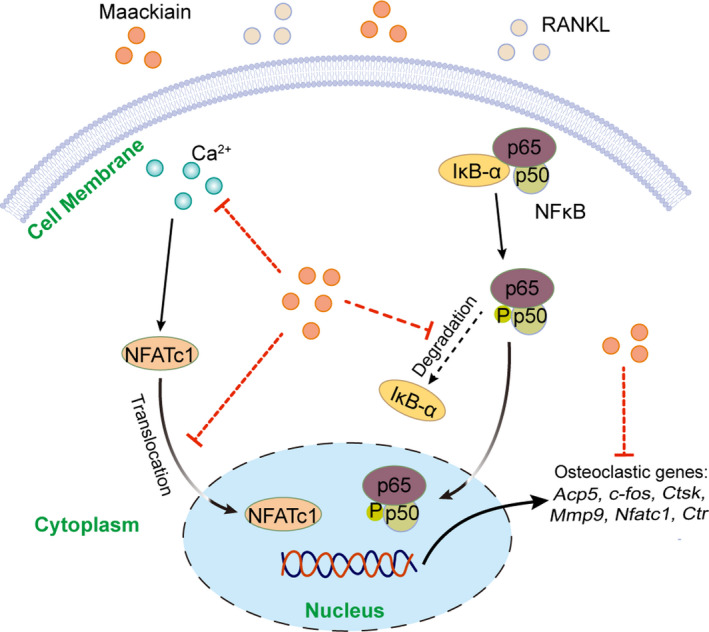
Proposed model showing the suppressive mechanisms of Maackiain in RANKL‐activated NF‐κB and NFATc1 signalling pathways

NFATc1 is the master transcription factor for the initiation of the transcription of osteoclastic genes.^9^ This study indicated that Maackiain inhibits the RANKL‐stimulated protein levels and transcriptional activity of NFATc1. In addition, the intensity of Ca^2+^ flux is enhanced under RANKL stimulation, resulting in the activation of calcineurin protein and downstream auto‐amplification of NFATc1.^15^ Notably, our results confirmed that Ca^2+^ oscillation levels are also suppressed by Maackiain, which further down‐regulates the expression of osteoclast‐related genes. Therefore, we concluded that Maackiain could inhibit the formation and resorbing function of osteoclasts via inhibiting NF‐κB signalling pathway and NFATc1 activity which results from blocking Ca^2+^ signalling pathway (Figure 6). These results indicated that Maackiain could be a potential drug against osteoclast‐related disorders.

## CONFLICT OF INTEREST

The authors declare that no conflict of interests exists.

## AUTHOR CONTRIBUTIONS

**Yuhao Liu:** Data curation (equal); Formal analysis (equal); Funding acquisition (equal); Methodology (lead); Software (equal); Visualization (equal); Writing‐original draft (equal). **Weizai Zeng:** Conceptualization (equal); Data curation (equal); Formal analysis (lead); Methodology (equal); Resources (lead); Software (equal); Visualization (equal); Writing‐original draft (equal). **Chao Ma:** Data curation (equal); Methodology (equal); Writing‐original draft (equal). **Ziyi Wang:** Methodology (equal). **Chao Wang:** Methodology (equal). **Shaobin Li:** Data curation (equal); Software (equal); Visualization (equal). **Wei He:** Conceptualization (equal); Data curation (equal); Resources (equal). **Qingwen Zhang:** Conceptualization (equal); Data curation (equal); Resources (equal). **Jiake Xu:** Conceptualization (equal); Funding acquisition (equal); Supervision (equal); Writing‐review & editing (equal). **Chi Zhou:** Conceptualization (equal); Supervision (equal); Writing‐review & editing (equal).

## Data Availability

The data that support the findings of this study are available from the corresponding author, upon reasonable request.
